# Medical Apps and the Gray Zone in the COVID-19 Era: Between Evidence and New Needs for Cybersecurity Expansion

**DOI:** 10.3390/healthcare9040430

**Published:** 2021-04-07

**Authors:** Giovanni Maccioni, Daniele Giansanti

**Affiliations:** Centre Tisp, Istituto Superiore di Sanità, 00161 Rome, Italy; gvnnmaccioni@gmail.com

**Keywords:** eHealth, medical devices, digital health, mHealth, cyber-risk, pacemaker, artificial pancreas, app, regulation, wearable device

## Abstract

The study focuses on emerging problems caused by the spread of medical apps. Firstly, it reviews the current role of cybersecurity and identifies the potential need to widen the boundaries of cybersecurity in relation to these apps. Secondly, it focuses on the pivotal device behind the development of mHealth: the smartphone, and highlights its role and current potential for hosting wearable medical technology. Thirdly, it addresses emerging issues regarding these apps, which are in a gray zone. This is done through an analysis of the important positions of scholars, and by means of a survey report on the increased use of various categories of apps during the COVID-19 pandemic, highlighting an accentuation of the problem. The study ends by explaining the reflections and proposals that emerged after performing the analysis.

## 1. Purpose of the Prospective Study

The proposed study is based on problems identified some time ago in relation to medical apps with regard to correct use by both the citizen and the medical actor, the clear identification of the intended use, and quality control and certification (when necessary).

As a prospective study, the first objective is to review the current role of cybersecurity and identify new needs to be covered in these medical apps.

The second objective is to highlight the opportunities of the smartphone device in mHealth, which has become a medium for wearable medical technology through dedicated apps and appropriate sensors.

The third objective, without the aim of performing a review, is to highlight the main problems found on these medical apps by the research world, which are considered to be in a gray zone.

The fourth objective is to highlight how the COVID-19 pandemic has exacerbated these problems. This is achieved through the development and submission of a targeted survey to investigate the increase in the use of these apps during the pandemic.

In line with the highlights of the Special Issue “Cybersecurity and the Digital Health: An Investigation on the State of the Art and the Position of the Actors” [1], this study ends with the expression of an opinion on how these issues are to be addressed (in particular with regard to how cybersecurity should act on these issues) and the role and positions that the various actors should have in order to act effectively in relation to the problem. It is in fact basic to understand [1] whether and how it is appropriate to expand and better generalize the role of cybersecurity in new border areas of the health sector, for example, with regard to nonmedical apps that can be confused with medical devices and for which noncompliant use could put patient safety at risk, especially during the COVID-19 pandemic. From a general point of view, this contribution aims to respond to this.

## 2. The Boundaries of the Cybersecurity Today and the Gray Zone of Medical Apps

### 2.1. The Boundaries of the Cybersecurity

Cybersecurity has applications in four main areas of the cybersystem, and can be used either in complex medical devices and/or complex interoperable and heterogeneous systems (involving elaboration systems, informatics, biomechatronics, bioengineering, electronics, networks, eHealth, and mHealth [1]). These four areas are data preservation, data access and modification, data exchange, and interoperability and compliance. The following systems have cybersecurity issues in health care:

#### 2.1.1. Wearable Medical Devices

Wearable medical devices [2,3,4,5,6], particularly implantable ones, are part of a heterogeneous system (e.g., pacemakers, artificial pancreases). In these systems, the wireless connection creates an environment that is potentially susceptible to cyberattacks.

#### 2.1.2. Picture Archiving and Communication Systems

Picture Archiving and Communication Systems (PACSs) [7] represent a form of medical device software (defined by the FDA as a Class II medical device) that is dedicated to the management of a diagnosis reached using medical imaging. A PACS embeds several parts such as elaborators, workstations, digital databases, digital data-stores, and digital applications that are subject to potential cyberattacks.

#### 2.1.3. Health Networks

As is well known, hospital companies today are strongly reliant on digital technologies. The cyber-risk is rapidly increasing with [1,7]:

1. The so-called dematerialization of administrative processes; and
2. The increased dependence on computerized biomedical and nonbiomedical technologies.

Hospital Information Systems (HIS) have been attacked and breached in some cases in terms of both privacy and activities [7].

### 2.2. The App in Health Care: The Gray Zone

Today, we are witnessing a diffusion in the market of apps that in some way have a correlation with aspects relating to health, in particular:

- *Apps certified as medical devices;*
- *Apps not certified as medical devices, whose manufacturers have decided by choice not to follow articulated certification processes, but which in any case have the potential to provide consistent physiological parameters;*
- *Apps that do not require certification based on intended use;*
- *Noncertified apps that have an intended use that would require a certification process and that do not have the potential to provide consistent physiological parameters.*

There is no doubt that in medical use, strict regulations and protocols that have broad implications ranging from diagnostics to therapy to legal aspects must be respected.

The way these apps are used (for example in telemonitoring or telemedicine) therefore has important implications that must be seriously considered.

At the moment, these implications do not seem to fall completely within the boundaries of cybersecurity, and include aspects of cybersecurity that are oriented towards the *cybersafety* of the patient and citizen, with strong correlations with market surveillance. A Google search with the words “Best Apps Health” returns millions of sites that support certain apps. However, this can disorient the patient and/or ordinary citizen when they face with this. It is therefore evident how a strong response is needed.

## 3. The Smartphone: New Opportunities as a Wearable Device Today in mHealth

Before the development of the smartphone, the monitoring and sending of physiological parameters took place through specially developed wearable devices, and the worlds of cell phones and telemonitoring remained separate [8].

As we know it today, the smartphone, with its development since 2008, has established itself first as a mediator between these two worlds and then consequently as a pivotal tool in mHealth for monitoring parameters, i.e., wearable with both sensor and processing potential. In general, the smartphone as [9] we know it today differs from the mobile phone due to the simultaneous presence of the following features:

1. The increased memory, a higher computing capacity, and a much more advanced data connection capacity due to the presence of dedicated operating systems;
2. A great potential for the production and management of multimedia content, such as taking high-resolution photos and producing video clips;
3. The ability to easily install free and/or paid features and/or applications (apps);
4. The provision of a high-resolution touch screen;
5. The possibility of using/maneuvering a virtual keyboard to interact with the various functions of the device (from the address book to the notepad), with the web, with the various applications installed, and with the so-called social networks;
6. Integration with sensors such as accelerometers, gyroscopes, magnetometers, thermometers, and even, in the most advanced models, photoelectric sensors, depth laser sensors, hall effect sensors, proximity sensors, and barometers;
7. The possibility of tethering (i.e., providing internet access to other devices such as access points) over the wireless network, e.g., Wi-Fi or Bluetooth, to devices such as other smartphones or mobile phones, laptops, or fixed computers;
8. The availability of GPS sensors.

In parallel to the development of the smartphone, dedicated operating systems have been spreading, some have consolidated (Android and IOS), while others have gone into obsolescence (Windows for example). Other new operating systems are emerging that also offer compatibility with consolidated operating systems; an example of this is the Harmony operating system from Huawei (Shenzhen, China), which seems to offer compatibility with Android.

Since the development of these devices and related devices, so-called virtual stores have proliferated, from which one can extract dedicated free and/or paid apps. Today, the most famous of these are connected to the two dominant OSs: Google Play (for Android) and App store (for IOS).

Initially, smartphones were not equipped with apps for health purposes.

Today, some smartphones already come with preinstalled apps for this purpose, which generally allow:

(1) Monitoring of some physiological parameters and activities related to wellness and fitness;
(2) Compatibility with other third-party apps and/or other sensors (piloted through the app) and/or smartwatches.

The examination of these case studies goes beyond the scope of this work, which is certainly not aimed at finding the best solutions in this regard.

Two well-known examples are the Samsung smartphone health app [10] on the Android operating system and the iPhone health app on the IOS operating system [11].

Harmony for Huawei, the new operating system that was previously mentioned, is also moving in this direction [12].

Recently, as a result of COVID-19, we have also seen a further push to search for new solutions and to verify the reliability of the physiological parameters provided by smartphones.

An noteworthy example is one of the most important physiological parameters considered in the pandemic: pulse oximetry [13]. Pulse oximetry is used to assess the severity of COVID-19 infection and to categorize the risk. Browne et al. [14]: (a) highlighted that over 100 million Samsung smartphones that contain dedicated biosensors (Maxim Integrated Inc, San Jose, CA) and preloaded apps to perform pulse oximetry are in use globally; and (b) successfully tested the Samsung S9 smartphone to determine if this integrated hardware meets the full FDA/ISO requirements for clinical pulse oximetry [14].

## 4. The Gray Zone of Apps That Provide Physiological Parameters and/or Suggest Therapies and/or Medical Support

In addition to any basic equipment that may concern health aspects, smartphones can be populated with apps for monitoring physiological parameters, medical support, and medical therapy.

It is now possible to find all kinds of apps in virtual stores. There are so many that regulation has become particularly complex, especially in the medical field. As far as we are concerned and in line with the objectives of the Special Issue “Cybersecurity and the Digital Health: An Investigation on the State of the Art and the Position of the Actors” in the journal *Healthcare* [1], this is the area that is most worrying. For this reason, we must pay attention to apps confounding the citizen and/or physician with respect to the related use [15].

App stores are now full of apps that can confuse the citizen.

The world of research has mobilized and has begun to address the problem of the quality and reliability of these apps with reference to all the players. It is in fact possible, for practically every medical sector, to find a great many reviews of such apps.

In the following, in line with the objectives of the study, we report some converging outcomes, regardless of the topic under consideration. Jones et al. focused on plastic surgery apps [16] and their review found that most applications with a medical purpose were not certified as a medical device, had not been validated in any peer-reviewed research, and did not have any documented involvement of medical professionals. They concluded that the potential consequences of such applications operating incorrectly are stark and represent a risk to patient safety. Trecca et al. [17] focused on otolaryngology apps and found that the apps that are currently available need further development and further dialogue between physicians and patients, and that formal support from professional and scientific associations should be encouraged. Knitza et al. analyzed German rheumatology apps [18] for patients and physicians available in German app stores and found a lack of supporting clinical studies, use of validated questionnaires, and involvement of academic developers. They concluded that to create high-quality apps, closer cooperation led by patients and physicians is vital. Tabi et al. [19] reviewed apps for medication management and identified detailed characteristics of the existing apps with the aim of informing future app development. They highlighted the need for improved standards for reporting on app stores and underlined the need for a platform to offer health app users an ongoing evaluation of apps by health professionals and other users and to provide them with tools to easily select an appropriate and trustworthy app. Haskins et al. published a systematic review of smartphone applications for smoking cessation [20]. Adhering to the Preferred Reporting Items for Systematic Reviews and Meta-Analyses (PRISMA) guidelines, apps were reviewed in four phases, in which they: (1) identified apps from the scientific literature; (2) searched app stores for apps identified in the literature; (3) identified top apps available in leading app stores; and (4) determined which top apps available in stores had scientific support. They highlighted that among the top 50 apps suggested by each of the leading app stores, only two (4%) had any scientific support.

Mandracchia et al. published a review [21] regarding mobile phone apps for food allergies or intolerances in app stores. They used the mobile app rating scale. They found that the included apps should be tested in trials and identified some critical points that can help improve the innovativeness and applicability of future food allergy and intolerance apps. Xie et al. reviewed cardiovascular disease mobile apps [22] and found that they are insufficient in providing comprehensive health information, high-quality information, and interactive functions to facilitate self-management. They concluded that: (a) end users should exercise caution when using existing apps; (b) health care professionals and app developers should collaborate to better understand end users’ preferences and follow evidence-based guidelines to develop mHealth apps. Nicholas et al. focused on psychiatry apps and, in particular, mobile apps for Bipolar Disorder (BD) [23]. In their systematic review they found that, in general, the content of the currently available apps for BD is not in line with practice guidelines or established self-management principles. Apps also fail to provide important information to help users assess their quality, with most lacking source citation and a privacy policy. Therefore, they conclude that: (a) both consumers and clinicians should exercise caution with app selection; (b) while mHealth offers great opportunities for the development of quality evidence-based mobile interventions, new frameworks for mobile mental health research are needed to ensure the timely availability of evidence-based apps to the public.

Huckvale et al. reviewed apps for asthma self-viewed management [24] and found that: (a) no apps for people with asthma combined reliable comprehensive information about the condition with supportive tools for self-management; (b) health care professionals considering recommending apps to patients as part of asthma self-management should exercise caution, recognizing that some apps may be unsafe. The COVID-19 pandemic itself has led to the development of a great many apps in the medical field. This development only further highlights the need for greater attention to the phenomenon. Ming et al. reviewed COVID-19 mobile health apps launched in the early days of the pandemic [25]. They highlighted that: (a) it can be difficult for health care professionals to recommend a suitable app for COVID-19 education and self-monitoring purposes; (b) it is important to evaluate the contents and features of COVID-19 mobile apps to guide users in choosing a suitable mobile app based on their requirements.

In light of the above, it is clear to see that we are witnessing the phenomenon of a gray zone in relation to these apps.

On the one hand, there are the needs of the actors in health processes, from doctors to citizens and, on the other hand, we have the rightly strict and rigorous rules of certification bodies such as the FDA.

Looking online, we are witnessing a proliferation of offers of incredible solutions. The scholars that analyze these offers with scientific rigor conclude that these solutions often represent a wild west [16]. Furthermore, these offers often do not adhere to the reality and what is published online is practically never supported by adequate scientific documentation and/or proof of what is being declared [16,17,18,19,20,21,22,23,24,25].

## 5. Highlighting the Problem during the COVID-19 Pandemic through an Electronic Survey

Examples that both intrigue and worry, when looking at the laypeople, especially those left alone in the COVID-19 era, are those represented by apps published online that promise solutions at your fingertips.

There are a multitude of apps that presumably saw an increase in popularity due to isolation, for example those that:

(a) Promise weight loss through dietetic programs;
(b) Promise to help you quit smoking;
(c) Promise to aid in the gym and/or with pseudo-rehabilitation motion programs;
(d) Support a fitness regime;
(e) Promise certain types of therapy (for example, psychological).

It is clear that the citizen when confronted with such an offer can become confused, relying on the app, and not seeking the advice of experienced professionals.

The COVID-19 pandemic has left many citizens isolated and lonely at home, so the problem has been further accentuated. On the one hand, there was a great opportunity for eHealth and mHealth to support the citizen; on the other, the offers available were very broad and often unclear. Many individuals have started to exercise, follow diets, quit smoking, etc. by relying on these apps in a self-taught way.

Herein, we have reported a few examples; however, by browsing the virtual stores, it is clearly evident that the problem is considerable and certainly worthy of attention. We developed a survey using established methods of electronic survey development, offering the opportunity to provide a useful measure of the acceptance and/or opinion of the citizen actor. Moreover, this modality, in the middle of the COVID-19 pandemic, allows for the maintenance of social distancing.

Recently, using social networks, we submitted an anonymous survey to a sample of 1150 young subjects; among them 1122 agreed to participate. The sample is represented by:

- Men: 580; with an average age of 25.7 years; a maximum age of 30 years; a minimum age of 19 years; a minimum of secondary school level education;
- Women: 542; with an average age 25.4 years; a maximum age of 30 years; a minimum age of 18 years; a minimum of secondary school level education.

The submission is still active and datamining will be further expanded. Here, with the aim of the study, we present the outcome on the first sample.

**Figure 1:**
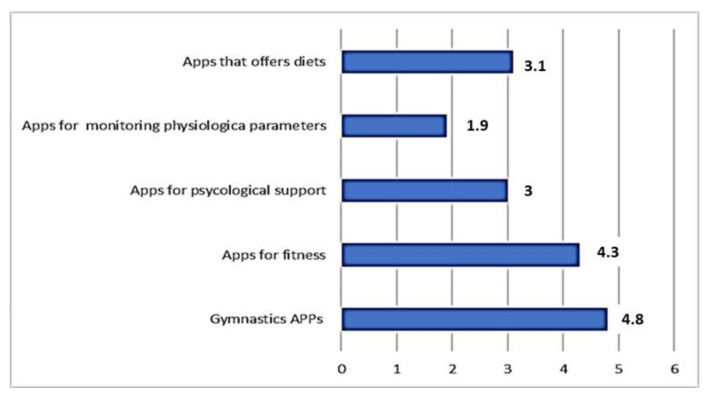
The answers to the Likert A question.

Figure 1 shows the answers to the Likert A question: “Please indicate the intensity of use of the following apps during the COVID-19 pandemic.”

For each subquestion of the Likert question, it was possible to assign a score from 1 (for nothing or never used) to 6 (very large use). Therefore, the threshold of average use (TA) was set at 3.5.

**Figure 2:**
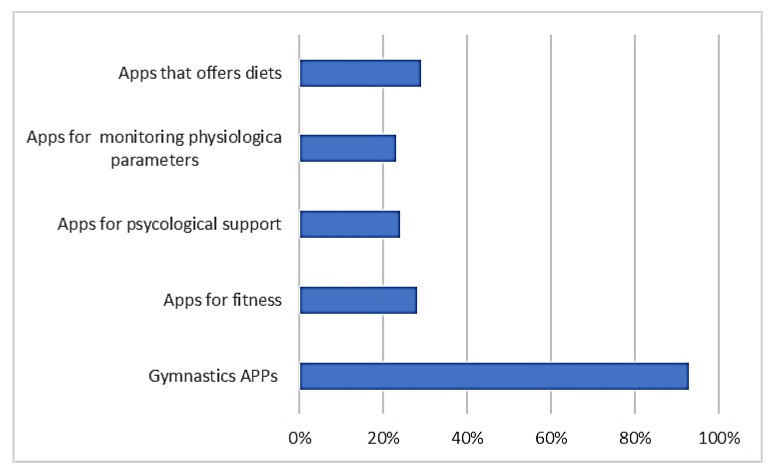
The answers to the Likert B question.

Figure 2 shows the answers to the Likert B question: “For the same Apps, indicated in the previous question, indicate the percentage increase compared to the period preceding the outbreak of the pandemic”.

For each subquestion of the Likert question, it was possible to assign a percentage according to these indications (0%, 5%, 10%, … 100%, more than 100%).

The Likert A question results highlight that:

- All the apps proposed have on average recorded use with an average score greater than 1;
- The apps for gymnastics and fitness had an average use greater than 3.5 = TA;
- Psychological and dietary therapeutic apps were also used, even if the score was not higher than TA.

The Likert B question results highlight that, in general, the average increase in use was always higher than 23%. Gym apps saw an increase in usage of over 90%. It is clear that before the pandemic, these apps had very little use when gyms were open and exploded during the pandemic.

A basic question was “In general, do you think that you have such knowledge of these Apps that allows you to distinguish the difference between medical apps and non-medical Apps?” This question could be answered with an evaluation ranging from one star (no knowledge) to six stars (a lot of knowledge). Therefore, the threshold of average use (TA) was set at 3.5. The average value obtained was 1.4, indicating a very low perception of knowledge << TA.

## 6. Conclusions

### 6.1. Highlights of the Study

Thanks to the astonishing development of mobile technologies, we are witnessing an enormous boom in mHealth technology.

This development is today conveyed by the smartphone device, which has gradually allowed the integration of wearable technology that once needed separate solutions [8].

Today, smartphones arrive configured with apps dedicated to health and with sensors that have the potential to provide measurements of important physiological parameters, such as oxygen saturation [14,15]. This applies to both of the dominant operating systems (Android and IOS) [10,11], and to new operating systems under development such as Harmony OS [12]. At the same time, apps dedicated to medical use are developed with the possibility of connecting to sensor devices and/or device kits.

This development has been so disruptive that it makes accurate regulation difficult if not impossible: some have defined it as regulating infinity [26]. The offer of these apps is impressive. For example, type “best App cardiology en” and you are diverted to a huge number of blog sites that promise you a list of the best cardiology apps. This is why the problem is relevant.

Currently, cybersecurity has well-defined and identified boundaries; these are more identified by concepts that revolve around the security of IT systems and data through solutions that prevent malicious attacks or the clumsy actions of operators [1].

Here, as regards apps that somehow fall into the sphere of medical applications, the concept of safe use must be understood with a different and broader meaning.

First of all, it must be said that an app with a medical destination, as dictated by the intended use, must be certified according to strict experimental protocols, strict documentation management [15], and by resorting to certification bodies. In some cases, the developers choose for their own reasons not to certify their apps; however, they must be aware that a doctor and/or medical specialist using mHealth/eHealth solutions, for example in telemedicine, is strongly bound by regulations and obligations in the use of certified devices and must defer to certification bodies.

The study proposed herein was developed according to various lines of thought.

A first step was to reassess the current boundaries of cybersecurity [1] to take into account the new needs concerning medical apps that fall in the gray zone.

In a second step, the evolution of the pivotal device of the great development of mHealth was analyzed: the smartphone, which today: (a) in the factory configuration, includes applications for health and openings to third-party apps in this area; (b) includes important sensorial integrations that allow, at least potentially, for the reliable measurement of physiological parameters important in the COVID-19 era [13,14].

The third step focused on the gray zone of these apps and explored the positions of various scholars who have published reviews of important categories of these apps, highlighting relevant problems.

In summary, the following criticalities emerge from these studies [16,17,18,19,20,21,22,23,24]:

- Most applications with a medical purpose were not certified as a medical device, had not been validated in any peer-reviewed research, and did not have any documented involvement of medical professionals; therefore, the potential consequences of such applications operating incorrectly are stark and represent a risk to patient safety;
- Most apps that are currently available need further development and dialogue between physicians and patients, and formal support from professional and scientific associations should be encouraged;
- There is a lack of supporting clinical studies, the use of validated questionnaires, and involvement of academic developers;
- To create high-quality apps, closer cooperation led by patients and physicians is vital;
- There is a low general level of scientific support;
- Apps are insufficient in providing comprehensive health information, high-quality information, and interactive functions to facilitate self-management;
- In general, the content of currently available apps is not in line with practice guidelines or established self-management principles.

Concerning the merits of the fabulous development of apps during the COVID-19 pandemic, the overall opinion is similar. It is clear [25] that it can be difficult for health care professionals to recommend a suitable app for coronavirus disease (COVID-19) education and self-monitoring purposes and that it is important to evaluate the contents and features of COVID-19 mobile apps to guide users in choosing a suitable mobile app based on their requirements.

After identifying various categories of apps of evident potential use during these great periods of isolation, the fourth step involved presenting a survey, the results of which generally highlighted both an increase in the use of these apps compared to the prepandemic period and a general lack of knowledge of the medical aspects in relation to their use. It is clear that an increase in the use of these apps combined with a lack of knowledge of the information aspects only reaffirms a potential increase in the problems highlighted above.

### 6.2. Final Reflections

It is evident that the problems that have emerged require wide-ranging and articulated solutions. Surely, the role of cybersecurity could be expanded and rethought to support solutions to the problems that have emerged. Many tools are available.

Tools to highlight the validity of an app exist, such as the mobile app rating scale [21].

Tools and methodologies that allow a community engaged approach to develop robust apps with close collaboration between potential users and developers also already exist [15].

Acceptance techniques through dedicated surveys such as the Technology assessment model (TAM) have already shown robustness [27]. Accessible databases through which to check the presence of certified apps exist and there is the possibility of creating online registers for apps that have followed qualification and validation paths, as was suggested by various authors [19]. However, it is extremely necessary to understand both: (a) why today some developers of these applications are not interested in having them validated by specialized entities in the medical, fitness, or nutrition fields; and (b) why there is an apparent poor lack or no interest from some of these entities in analyzing and validating that the applications meet the necessary requirements, e.g., that they do not harm the physical or mental health of users. All this is important and serves to explain why we are witnessing a wild west in the field of App production, and why we are seeing a proliferation of blog sites, that in some cases seem to support this or that App as if they were a cooking product.

In all probability, the answer must be sought in the pressures of the market: the same pressures that should guarantee better control, from clearer and more understandable indications in app stores, to surveillance policies and blanket monitoring of these apps in order to stimulate entities and developers to better collaborate, to the intensification of the supply and demand of support and services from development to training. It is evident that cybersecurity can help us, but it must be reassessed to include a concept of security that is not merely IT, and is more an expansion of citizen safety. Cybersecurity can certainly intervene in various ways, for example:

1. Through monitoring policies and regulation initiatives;
2. Through citizen training and information policies, perhaps offered in an e-learning format.

The first initiative should start from the supranational surveillance and regulation of virtual stores for all operating systems. It should also include the monitoring of public sites and supporting the advertising of platforms with certified apps and/or those that have followed a qualification path. It should ensure that the developers are motivated to follow these paths as well as the entities that offer the services.

The second initiative should also begin in virtual stores with the inclusion of clear and understandable information, not only to health care professionals but to laypeople. It should then continue with initiatives that involve school-aged citizens that help them understand and explore the situation [28].

## Data Availability

Data sharing not applicable.
